# Influence of Days in Milk and Parity on Milk and Blood Fatty Acid Concentrations, Blood Metabolites and Hormones in Early Lactation Holstein Cows

**DOI:** 10.3390/ani10112081

**Published:** 2020-11-10

**Authors:** Quynh Chau Dang Van, Emilie Knapp, Jean-Luc Hornick, Isabelle Dufrasne

**Affiliations:** 1 Institut Supérieur Industriel Agronomique, Haute Ecole Charlemagne, 3 Rue Saint-Victor, B-4500 Huy, Belgium; quynh.dangvan@hech.be; 2 Nutrition Unit, Department of Animal Production, Faculty of Veterinary Medicine, University of Liège, 20/B43 Boulevard de Colonster, B-4000 Liège, Belgium; eknapp@student.ulg.ac.be (E.K.); jlhornick@uliege.be (J.-L.H.)

**Keywords:** dairy cow, metabolism, milk, negative energy balance, peripartum

## Abstract

**Simple Summary:**

At the onset of lactation, the energy intake is not sufficient to meet the high energy requirement due to milk production increase. Consequently, body reserve is mobilized to support this negative energy balance situation. Severe negative energy balance can lead to health and production concerns. Certain blood and milk parameters could be used as indicators of lipomobilization and can thus predict the energy status in postpartum cows. Therefore, the objective of this paper was to study the influence of physiological factors that affect the energy balance, such as lactation stage and parity, on blood and milk parameters in healthy early lactation cows. Thirty-two Holstein dairy cows from five Belgian commercial farms were followed during two calving periods. This study confirms that lactation stage along with energy balance and parity significantly influence blood and milk parameters.

**Abstract:**

The objective of this paper is to study the influence of physiological factors that affect the energy balance, such as lactation stage and parity, on milk yield and composition, milk and blood fatty acid concentrations, blood metabolites and hormones in healthy early lactation cows. Thirty-two Holstein dairy cows from five Belgian commercial farms were followed. The grass silage-based diets fed to cows fell within normal composition ranges typically offered to dairy cows on commercial dairy farms in the region. Milk and blood were sampled at each official milk recording and used for the determination of milk fat and protein, milk and blood fatty acids, blood metabolites and hormones concentrations. The considered period was 7 to 150 days in milk. As lactation progressed, concentrations of milk 18:0 and 18:1c9, as well as blood non-esterified fatty acids and β-hydroxybutyrate, decreased, and those of milk C4–C14, blood cholesterol, triglycerides, insulin and IGF-I increased, agreeing with the extensive mobilization of body reserves in early lactation. Lower concentrations of milk C4–C14 and 16:0 and concomitant higher concentrations of milk 18:0 and 18:1c9 suggest a larger body reserve mobilization in first parity cows compared with greater than or equal to second parity cows. This study confirms that early lactation stage along with parity significantly influence milk fatty acids, such as 18:1, and blood metabolites and hormones, such as NEFA and insulin.

## 1. Introduction

The peripartum period is essentially critical for dairy cows. Energy requirement increases at the onset of lactation. Energy intake is not sufficient to meet this high energy requirement due to the increase in milk production resulting in a physiological negative energy balance (NEB). This NEB can lead to health disorders in some cases. Energy balance is thus associated with days in milk (DIM), parity, milk yield, milk fat and protein concentrations, body condition score (BCS) and metabolic parameters such as non-esterified fatty acids (NEFA) and β-hydroxybutyrate (BHB) [1].

A large amount of epidemiological data has shown that increased blood concentrations of NEFA and BHB are associated with negative health and production outcomes at both individual cow and herd level [2,3,4]. The peripartum circulating concentration of NEFA reflects the magnitude of body reserve mobilization and mirrors DMI [5], while BHB, the predominant ketone body in blood, reflects the completeness of oxidation of fat in the liver [3]. As the supply of NEFA to the liver exceeds its ability to completely oxidize the fatty acids (FA) to supply energy, the production of BHB and the risk of fatty liver and ketosis increases [1]. Several authors reported an abnormal blood NEFA concentration of >0.4 mmol/L between 7 and 10 days prepartum and of ≥1 mmol/L in multiparous cows between 3 and 14 DIM [3,6], and abnormal blood BHB concentrations of ≥1.2 mmol/L in the first or second week postpartum [2,3,6]. Blood BHB concentrations can be measured cow-side, whereas blood NEFA concentrations are usually measured in laboratory and are therefore associated with an important lag time compared with BHB measurement [3,4]. Moreover, both measurements are invasive, time-consuming and costly. Therefore, the search for fast, inexpensive testing methods that are more suitable in terms of animal welfare considerations has increased [7,8].

Because official daily milking recording is done in most dairy commercial farms, there is a growing interest in evaluating milk parameters as indicators of energy balance in early lactation. Researchers have focused on milk composition, using the ratio of fat to protein percentage as an indicator of lipomobilization and the NEB status in postpartum cows [1,9,10]. Cows with a milk fat-to-protein ratio in early lactation greater than 2.0 showed an increase in postpartum diseases such as retained placenta, left displaced abomasum or metritis [1,10]. However, this ratio does not take the variability in different milk FA concentrations into account. Because the increase in certain milk FA (predominantly c9-18:1) originating from body reserve mobilization reflects the severity of NEB in early lactation, determining the FA composition of milk can predict energy status in early lactation cows [11,12]. Moreover, with the potential of mid-infrared spectrometry predictions to integrate milk FA composition directly in the official daily milking recording [13], the possibility of future routine use of this information has increased. Lower percentages of milk saturated FA (predominantly 10:0, 12:0, 14:0, 16:0) and higher percentages of mono-unsaturated FA (predominantly c9-18:1) were observed in early lactation (around 30 DIM) compared to mid-lactation (around 150 DIM), as NEB became less severe [8,12].

Currently, data on lactation profiles for milk yield and composition, milk and blood FA composition, and blood metabolites and hormones across physiological factors affecting the energy balance (such as lactation stage and parity) are scarce. Therefore, the objective of the present paper was to study the influence of DIM and parity on milk yield and composition, milk and blood FA concentrations, blood metabolites and hormones in healthy early lactation Holstein cows.

## 2. Materials and Methods

### 2.1. Farms, Animals, Diets and Management

This study was carried out on Walloon commercial farms where the animals were handled with care according to the Walloon good practices. Thirty-two Holstein dairy cows from five commercial farms were randomly selected. The only criterium was a ratio of 2 primiparous for 3 multiparous: more precisely there are 10 primiparous and 22 multiparous. Furthermore, no cows calved during the grazing period were selected to avoid effects of diet change in relation with grazing period. All the cows were healthy, without any problem at calving till 1 week post-partum. The calving period considered was from February 2010 until January 2011. There were no samplings during the grazing period. The farms were located in the province of Liège (Belgium) where the largest part of agricultural land is used for summer grazing and the production of grass silage for winter feeding. The size of the herd expressed as number of cows in lactation was 72 ± 6.7. The age at first calving was 27 ± 1.8 months and the calving interval was 397 ± 14.4 days. The first heat was observed 46 ± 16.2 days postpartum. The first artificial insemination was carried out 78 ± 17.1 days postpartum. Ovarian cysts were detected on 13 out of 32 cows. Cows were followed up for reproductive performances. At the end of the observation period (111 ± 21.4 days postpartum), all cows were pregnant. The annual milk yield (on a 305-day basis) was 7726 ± 1201 kg/cow with fat and protein concentrations of 43 ± 3.1 and 34 ± 1.0 g/kg, respectively. All animals were clinically healthy before the study, were vaccinated and dewormed according to classical schedules and had an infectious bovine rhinotracheitis-free status. All diets fed to cows fell within normal composition ranges typically fed to dairy cows on commercial dairy farms in the region. The grass silage-based diets (62 ± 10.3% of forages, dry matter (DM) basis) were balanced with purchased maize silage, pressed and ensiled sugar beet pulp, concentrates and vitamin–mineral mix to meet energy and protein requirements of cows according to the Dutch VEM/DVE-OEB feeding system [14,15] for a cow of 650 kg yielding 22 up to 29 kg of milk/day according to the average production of the herd (Table 1). Automatic dispensers were used to provide concentrates according to the individual milk production of cows.

### 2.2. Samplings and Measurements

Individual cow milk yield was recorded and sampled monthly on the day of the official milk recording organized by the Walloon Breeding Association (Ciney, Belgium). Milk samples were refrigerated at 4 °C for analyses.

Blood samples were taken monthly on the day of the official milk recording. The first blood sample was collected on the first official milk recording (i.e., between day 7 and day 30 postpartum) and the last (fourth) blood sample was collected on the day when pregnancy was diagnosed by echography or up to 150 days postpartum. Blood samples were collected via venipuncture of coccygeal vessels 2–5 h after the morning meal and placed in two sets of BD Vacutainer^®^ tubes, the first set containing no additive to monitor serum metabolites (serum tubes) and the second heparinized set to monitor plasma metabolites (plasma tubes). Serum tubes were left at room temperature until complete clotting (within 30 min), whereas plasma tubes were immediately put in ice. Serum and plasma tubes were centrifuged at 4 °C for 20 min at 1500× *g*. Serum and plasma were harvested and stored at −20 °C until analyses. An additional blood sample was collected in a 1 mL syringe and was tested for glucose and BHB concentrations using a cow-side device at room temperature within 15 min after sampling (before clotting).

Obstetrical examination and body condition scoring at a scale of 1–5, 1 being emaciated and 5 obese [16], were performed along with milk and blood samplings.

### 2.3. Analyses

A portion of each milk sample was used for fat and protein analyses by the mid-infrared spectroscopic method—adapted from the AOAC method 972.16—[17] using the MilkoScan FT6000 spectrophotometer (Foss Analytical, Hillerød, Denmark) at the certified Milk Control Station (Battice, Belgium; Belgian accreditation number 262-TEST in compliance with ISO 17025), and the rest of each sample was used for fat extraction.

The following analytical procedures for milk fat extraction and FA methylation were previously described in [18]. Milk fat was extracted following the method described by Hara and Radin [19]. Milk samples were centrifuged at 17,800× *g* at 4 °C for 30 min, and 300 to 350 mg of the fat cake was collected. Fat extraction was performed with 7.2 mL of a hexane/isopropanol (3:2, vol:vol) solution, followed by 4.8 mL of a Na_2_SO_4_ solution (67 g/L). The upper phase was removed and dried with 1 g of anhydrous Na_2_SO_4_. It was then transferred again and brought to dryness under a continuous stream of N_2_. Fatty acids were first hydrolyzed in a solution of KOH in methanol (0.1 M) at 70 °C for 60 min, then methylated in a solution of HCl in methanol (1.2 M) at 70 °C for 20 min, and finally extracted with hexane.

Milk Fatty acid methyl esters (FAME) were separated and quantified with a gas–liquid chromatograph (GC-2014, Shimadzu, Kyoto, Japan) equipped with a flame ionization detector, an automatic injector, and a fused silica capillary column (100 m × 0.25 mm i.d.), coated with a 0.2 μm film of biscyanopropyl polysiloxane (CP-Sil 88; Chrompack, Middelburg, The Netherlands). The chromatographic conditions employed were as follows: carrier gas, H_2_; injection volume, 1 µL; split-injection ratio, 1:50; injector temperature, 270 °C; detector temperature, 300 °C. In order to maximize the separation of FAME, the oven temperature program was adapted from [20,21]—the temperature used was 45 to 250 °C with a plateau at 175 and 215 °C. Each peak was identified and quantified by comparison of retention times with pure FAME standards (Alltech Associates Inc., Deerfield, IL, USA). Each FA was expressed as a percentage of identified FA.

Whole blood glucose and BHB concentrations were measured cow-side using a device validated in cattle (Precision Xtra^®^, Abbott, Berkshire, UK; [22]).

Serum total NEFA concentrations were determined by a photometric measurement (Randox NEFA, Crumlin, UK) using a clinical chemistry analyzer (Beckman Coulter, Krefeld, Germany).

Serum urea concentrations were determined by ultraviolet kinetic test (Urea OSR6134, Beckman Coulter, Krefeld, Germany) using a clinical chemistry analyzer (Beckman Coulter, Krefeld, Germany).

Serum cholesterol concentrations were determined by an enzymatic coloration test (Cholesterol OSR6116, Beckman Coulter, Krefeld, Germany) using a clinical chemistry analyzer (Beckman Coulter, Krefeld, Germany).

Serum triglycerides (TG) concentrations were determined by an enzymatic coloration test (Triglyceride OSR60118, Beckman Coulter, Krefeld, Germany) using a clinical chemistry analyzer (Beckman Coulter, Krefeld, Germany).

Plasma insulin and insulin-like growth factor-I (IGF-I) were determined using a radioimmunoassay kit.

FA in the serum NEFA were analyzed according to Husek et al. [23] who described a simple and rapid procedure for the extraction and methylation of individual free FA in serum. Gas–liquid chromatography analyses were performed as described above for FA in milk samples, except for the split ratio which was 1:2.5.

### 2.4. Statistical Analysis

Individual data were introduced in a database in which the cow/day was the unit. The data were compared according to classes based on DIM (7–50 vs. 51—99 vs. 100–150) and parity (1 vs. ≥2). The data are reported as least squares means ± standard error of the means and were analyzed using the proc mixed procedure of SAS (version 9.1.3; SAS Institute Inc., Cary, NC). The statistical model included the effects of DIM, parity, farm, cow/farm and random error. DIM, parity and farm were considered as fixed effects and cow/farm as random effect. Overall, differences between group means were considered to be significant when *p* <0.05. When a significant group effect was observed, the Tukey option was used to compare means.

## 3. Results

### 3.1. Influence of Days in Milk and Parity on Milk Yield and Composition and Fatty Acid Concentrations in Early Lactation Holstein Cows

The influence of DIM and parity on milk yield and composition and FA concentrations in early lactation Holstein cows is summarized in Table 2. Milk yield was ≥27 kg/d throughout the whole observation period and was affected by parity (*p* < 0.001), by DIM (*p* > 0.01) and by farm (*p* < 0.001). First parity cows had lower milk yields compared to greater than or equal to second parity cows (23.62 vs. 37.61 kg/d, respectively). The milk yield in the different farms ranged from 24.84 to 38.9 kg/d on average. Milk fat and protein concentrations were respectively over 42 and 30 g/kg throughout the whole observation period and were not affected by DIM or farm *p* > 0.05). The fat/protein ratio ranged from 1.36 to 1.45 and was not affected by DIM, or parity or farm (*p* > 0.05). The most prevalent milk FA values were saturated FA (SFA), with 16:0 being the most important. The unsaturated FA most represented in milk was monounsaturated FA (MUFA, predominantly 18:1c9), and polyunsaturated FA (PUFA) was the least represented. Overall, milk FA concentrations were both affected by DIM and parity. The lowest concentrations of milk C4–C14 and odd branched-chain FA (OBCFA; 15:0, 17:0, iso15:0, 17:0 and anteiso 15:0, 17:0) were observed during ≤50 DIM compared with the other two DIM groups (*p* < 0.05), whereas 18:0 and 18:1c9 concentrations were the highest (*p* < 0.05). Milk 16:0 was higher between 51 and 99 DIM (*p* < 0.05), and PUFA values were the lowest during this DIM period (*p* < 0.001). As for the parity, lower concentrations of milk C4–C14, 16:0 FA were observed in first parity cows compared with greater than or equal to second parity cows (*p* < 0.05), whereas 18:0, 18:1c9, OBCFA and PUFA concentrations were higher (*p* < 0.05). Only 16:0 and OBCFA concentrations were affected by farm (*p* < 0.05; Appendix A Table A1.).

### 3.2. Influence of Days in Milk and Parity on Blood Fatty Acid Concentrations, Metabolites and Hormones in Early Lactation Holstein Cows

The influence of DIM and parity on BCS, blood FA concentrations, metabolites and hormones in early lactation Holstein cows is summarized in Table 3. Cows showed a loss of BCS in the group 51–99 DIM (*p* < 0.001) and regained it after 100 DIM. There were no significant differences according to parity. Blood metabolites and hormones were affected by DIM. The lowest concentrations of urea, cholesterol, TG, insulin and IGF-I, but higher concentrations of total NEFA and BHB, were observed during ≤50 DIM compared with the other two DIM groups (*p* < 0.05). Except for 16:0, individual free FA concentrations in blood were affected by DIM (*p* < 0.05). The lowest concentrations of 18:0 and 18:2 were observed at ≤50 DIM compared with the other two DIM groups (*p* < 0.05), whereas the highest concentrations of 18:1c9 were observed at ≤50 DIM. A significant effect of the parity was observed on IGF-I and 16:0. A lower concentration of 16:0 was observed in first parity cows compared with greater than or equal to second parity cows (*p* < 0.05), whereas the IGF-I concentration was higher (*p* < 0.05). The farm effect was significant on almost all these variables, except on BCS, glucose and BHB concentrations (Appendix A Table A2).

## 4. Discussion

### 4.1. Milk Yield and Composition

The milk yield was affected by DIM and averaged 30.65 kg/d throughout the whole observation period. This higher milk yield observed during the first 100 days of lactation may be attributed to good milk production sustainability due to genetic merit. As we expected, there were significant differences between farms due to management difference. Several authors showed that cows with a fat-to-protein ratio greater than 2.0 in early lactation were more likely to suffer from postpartum diseases [1,10]. In the present study, the fat-to-protein ratio was far below this threshold and ranged from 1.36 to 1.45. However, to use milk fat and protein information as predictors of the metabolic diseases that commonly occur within 30 DIM, samples should be evaluated within nine days postpartum [10], which was not the case in our study. Milk fat and protein concentrations were not affected by DIM or parity in contrast to milk FA concentrations, suggesting that individual milk FA concentrations should be taken into account to explain the variability in the energy status of cows.

### 4.2. Milk Fatty Acid Concentrations

In the present study, mean SFA (predominantly C4–C14, 16:0 and 18:0) was 64.78% of the identified FA vs. 25.90% for MUFA (predominantly 18:1c9) and 2.51% for PUFA. These data are in accordance with those reported by Grummer [24] for a typical milk fat from a dairy cow (70% SFA, 25% MUFA and 5% PUFA, including n-3 FA).

Lactation stage along with energy balance significantly influences milk FA composition [25,26]. The common NEB during the first weeks after parturition in dairy cows is compensated by the mobilization of fat from body reserves. resulting in the release of preformed FA; 18:1c9 is the predominant unsaturated FA in adipocytes and is primarily released through lipolysis during NEB [27]. Subsequently, pre formed long-chain NEFA (≥C18) derived from plasma are incorporated into milk fat and inhibit the de novo synthesis of short-chain FA (C4–C14) by the mammary gland [28]. Therefore, blood NEFA concentrations are in relation with milk long-chain FA concentrations. As lactation progresses, concentrations of preformed FA decrease, and those of de novo FA and mixed-origin FA (16:0) increase [25]. In the present study, the highest concentrations of 18:0 and 18:1c9, and the concomitant lowest concentrations of C4–C14, during ≤50 DIM compared to the other two DIM groups indicate an increase in body reserve mobilization. By contrast, the short-chain FA proportion was the lowest in the ≤50 DIM group; these FA are synthetized de novo synthesized in the mammary gland from acetate produced in rumen (linked to DMI and microbial activity). These results are consistent with the results of Gross et al. [12], Nogalski et al. [29] and Vrankovic et al. [8]. Because of its double origin from de novo synthesis and circulating blood NEFA, 16:0 did not show the same pattern as 18:0, 18:1c9 or C4–C14.

Jorjong et al. [30] showed that early lactating cows with ≥24% of milk 18:1c9 during the second week of lactation had about a 50% chance of having blood plasma NEFA concentrations of ≥0.6 mmol/L during the first eight weeks of lactation. This NEFA threshold of 0.6 mmol/L is most commonly reported in the literature [30,31,32]. Beyond this threshold, the risks of negative impacts on health and on reproduction are increased [3]. In the present study, throughout the whole observation, milk mean concentrations of 18:1c9 were ≥24% of the identified, and FA and blood NEFA concentrations remained at <0.6 mmol/L. Only three samplings were beyond 0.6 mmol/L, representing less than 10% of the total observation number. This suggests that there are no risks of negative impacts on health or reproduction in the sampled group.

Milk linear odd-chain FA (15:0 and 17:0) might provide information on the cow’s glucose status, as they are de novo synthesized from propionate by rumen bacteria or in the mammary gland [33]. Consistent with this statement, in the present study, milk OBCFA tended to increase as lactation progressed.

As for the influence of parity on milk FA concentrations, the literature data are inconsistent. Berry et al. [34] showed that first parity cows were lighter, lost more BCS in early lactation, had lower net energy intake and were in NEB for longer compared with greater than or equal to second parity cows, whereas Friggens et al. [35] showed that parity one cows mobilized significantly less than parity two and three cows. In the present study, lower concentrations of C4–C14 and C16, and concomitant higher concentrations of 18:0 and 18:1c9, in milk suggested a larger relative body reserve mobilization in parity 1 cows compared with ≥2 parity cows.

### 4.3. Blood Metabolites and Hormones

It has been shown that blood concentrations of NEFA and BHB, the most important markers of NEB, increase in early lactation [2,3,6]. The mobilization of body reserve during NEB results in elevated concentrations of circulating long-chain NEFA, which are intensively oxidized to acetyl-coenzyme-A to allocate energy but are also used by the mammary gland for milk fat synthesis. Therefore, blood NEFA concentrations parallel milk long-chain FA concentrations. As the supply of NEFA to the liver exceeds its ability to completely oxidize the FA from acetyl-coenzyme-A to citrate through the Krebs cycle and respiratory chain reaction, to make energy available for the body as ATP, acetyl-coenzyme-A is diverted to the production of ketones (predominantly BHB), eventually resulting in a status of ketosis, or NEFA can be esterified and stored in the liver as TG, eventually resulting in the status of fatty liver [36,37,38]. Although both blood NEFA and BHB are related to NEB, they cannot be used interchangeably [39]. NEFA concentrations do not determine the extent of incomplete oxidation and production of BHB [40]. Therefore, elevated concentrations of one should not be extrapolated to possible elevated concentrations of the other metabolite [4,39]. Moreover, van Knegsel et al. [37] hypothesized that part of the metabolic effect of NEB is an imbalance in the ratio of lipogenic and glucogenic nutrients in blood. Lipogenic nutrients originate from ruminal acetate and butyrate, and lead to the production of acetyl-coenzyme-A, whereas glucogenic nutrients originate from ruminal propionate or intestinal glucose and lead to the production of oxaloacetate. Acetyl-coenzyme-A and oxaloacetate are oxidized to form citrate that further proceeds through a series of reactions to generate energy to the body, as described above. Providing acetyl-coenzyme-A and oxaloacetate in a one to one ratio is ideal for efficient metabolism [37]. However, in early lactation, an excess of lipogenic nutrients (stimulated by body reserve mobilization) and a deficiency in glucogenic nutrients (driven towards the production of lactose) induce an imbalance in the ratio of lipogenic:glucogenic nutrients, which eventually limits ATP production [37]. Whereas the postpartum ketone production is a normal adaptive response to increased energy demand, excess ketone accumulation is abnormal and impairs production or health [2]. Some authors have suggested that hyperketonemia in early lactation (≤50 DIM) may start at levels of BHB >1.2 mmol/L [2,6,41] or >1.4 mmol/L [31,42]. As for blood NEFA, concentrations of >0.6 mmol/L were suggested for the detection of NEB and fatty liver in lactating cows [30,31,32,42], and concentrations considered as high were ≥1 mmol/L in multiparous cows between 3 and 14 DIM [3,5,6]. NEFA concentrations averaged <0.2 mmol/L in the late lactation and the dry period [32]. In the present study, BHB and NEFA concentrations were under these thresholds most commonly reported in the literature. The highest concentrations of total NEFA and BHB at ≤50 DIM compared to the other two DIM groups only indicate a mobilization of body reserve during NEB, resulting in a lower BCS between 51 and 99 DIM.

Kim and Suh [43] showed that the common NEB during the first weeks after parturition in dairy cows is closely related to lower serum total cholesterol concentrations. Sepulveda-Varas et al. [44] showed that cholesterol was negatively correlated with serum NEFA but not with BHB during the first two weeks after calving, suggesting that body reserve mobilization, NEFA and cholesterol concentrations in serum are interrelated. Compared to NEFA and BHB, these authors suggested that serum cholesterol may be a better indicator of energy balance status during early lactation [43,44]. In the study of Kim and Suh [43], serum cholesterol concentrations averaged 4.32 mmol/L during the first month postpartum, then increased gradually thereafter to 6.46 mmol/L at the fourth month for cows with marked body condition loss postpartum, whereas in the study of Sepulveda-Varas et al. [44], serum cholesterol concentrations ranged from 1.94 to 3.18 mmol/L during the first three weeks postpartum. In our study, the pattern of change in serum cholesterol concentrations in early lactation was consistent with the studies cited above; the lowest value was found for the ≤50 DIM group (4.01 mmol/L), then it increased thereafter to 4.95 mmol/L for the 51–99 DIM group, agreeing with the extensive mobilization of body reserve in early lactation.

Serum urea concentrations vary around the time of parturition [43,45] and might provide information on the supply of protein to the small intestine, which is linked to DMI. In our study, the pattern of change and data of serum urea concentrations in early lactation were consistent with other studies [43,45]; the lowest value was found for the ≤50 DIM group (3.15 mmol/L), which then increased as lactation progressed.

As discussed above, during the peripartum period, DMI is depressed, energy requirement is high, energy balance becomes negative, and TG stored in the body reserve is mobilized, resulting in increased NEFA in the blood. These NEFA are taken up by the mammary gland for milk fat synthesis, oxidized by the liver (and other tissues), or esterified to TG in the liver. Triglycerides can be exported from the liver as very low density lipoproteins (VLDL), but the rate of this process is limited in ruminants compared with other species [46]. Because phosphatidylcholine is required for VLDL assembly, the lack of sufficient dietary choline supply, coupled with the increased demand for methionine for milk synthesis, could render choline a limiting substrate for VLDL synthesis, further slowing the rate of TG export from the liver, which could contribute to the development of fatty liver and limit milk production [47]. Lower concentrations of TG were observed at ≤50 DIM (0.11 mmol/L) compared with the last DIM group (0.15 mmol/L), indicating a lower TG export from the liver. The values in our study are consistent with those in the studies of Kim and Suh [43] and Janovick Guretzky et al. [47]. However, in the study of Kim and Suh [43], the TG concentrations were not different from month 1 to month 4 of lactation between the two groups of cows enrolled in the study, and TG concentrations averaged 0.21 mmol/L.

In addition to blood NEFA and BHB, decreased blood glucose concentration is also an indicator of NEB in postpartum dairy cattle [5]. Some authors suggested that hypoglycemia in early lactation may start at levels of glucose ≤39.6 mg/dL [48]. Moreover, van Knegsel et al. [40] showed a negative relationship of plasma glucose with plasma BHB concentration. In the present study, blood glucose concentrations were above the threshold suggested by Dubuc and Buczinski [48]. However, glucose concentrations did not follow the pattern of change described in other studies [43]. Blood glucose is under tight homeostatic control, and therefore may be a poor analyte for monitoring or investigating health and production outcomes [36].

Hypoinsulinemia in dairy cows in early lactation is part of an adaptation process around the time of parturition in support of lactation. Low plasma insulin concentrations reduce glucose uptake by muscle and body reserves, and facilitate the increased uptake of glucose by the mammary gland, which is not insulin-responsive [4,36,49]. Moreover, van Knegsel et al. [40] showed a negative relationship between insulin and plasma NEFA, and confirmed that insulin plays a key role during NEB in early lactation. The liver is refractory to growth hormone (GH) during NEB, and this uncoupling of the GH–IGF axis results in dramatically reduced plasma concentrations of IGF-I [50]. In this way, in the present study, insulin concentration was the lowest in the ≤50 DIM group and was paralleled by lower concentration of IGF-I, compared with the other two DIM groups. Both hormones increased as lactation progressed. The lowest concentrations in IGF-1 observed in multiparous cows have been observed by other authors [40,49,50]. They can be explained by a difference in the control of body reserve mobilization between primiparous and multiparous cows, allowing nutrient partition into growth as well as milk in primiparous cows [51]. In our results, higher long chain FA in milk and higher IGF-1, associated with no difference in NEFA and BHB blood concentrations, suggest also a difference in energy metabolism.

### 4.4. Blood Fatty Acid Concentrations

In line with the study of Douglas et al. [52], 18:0, 18:1c9 and 16:0 were the predominant FA, and together these FA accounted for >90% of blood NEFA. The concentration of 18:2 in blood NEFA was low compared to the other FA because ruminants have a limited availability of PUFA due to rumen biohydrogenation and the limited synthesis of these PUFA by ruminal bacteria [53]. However, dairy cows have partially offset this limited availability by their very efficient intestinal absorption of PUFA [54]. Higher concentrations of 18:1c9 were observed at the beginning of the lactation period, which confirms that this FA is primarily released from adipocytes through lipolysis during NEB [27].

## 5. Conclusions

This study confirms that the lactation stage significantly influences milk FA, blood metabolites and hormones concentration. As lactation progresses, concentrations of milk 18:0 and 18:1c9, and blood NEFA and BHB, decrease, and those of milk C4–C14, blood cholesterol, TG, insulin and IGF-I increase. Individual milk FA concentrations, such as 18:1c9 and C4–C14, could be more suitable than blood NEFA and BHB measurements—which are invasive, time-consuming and costly—as indicators of the energy status of cows. The influence of parity on milk and blood profiles is more limited. Lower concentrations of milk C4–C14 and 16:0, and concomitant higher concentrations of milk 18:0 and 18:1c9 FA, could suggest a larger body reserve mobilization in first parity cows compared with greater than or equal to second parity cows. However, as NEFA is not modified by parity and IGF-1 is higher in primiparous cows, it seems there is a difference in the energy metabolism between multiparous and primiparous cows.

## Figures and Tables

**Table 1:** Composition of diets in the 5 farms.

Farm	1	2	3	4	5
composition in DM of a base diet in the partial total mixed ration (kg DM/cow)					
grass silage	12	10	8.5	10.3	12.5
maize silage	4.2	4.5	6.2	2.8	3.5
beet pulp silage	1.5		2		1
beet pulp dried	0.4			0.9	
concentrate (% CP)	3.3 (30%)	1 (25%)	1.4 (35%)	0.9 (35%)	1.8 (25%)
minerals	0.1	0.15	0.1	0.15	0.1
level of production permitted by energy and proteins according to the dutch system (kg)					
	30	19	22	23	26
production concentrate in automatic distributors, robot or in the milking parlor (kg DM/day and cow at 30 DIM)					
Concentrate (% CP)	5.4 (19)	6.3 (25)	4.5 (18)	5.8 (19)	4.5 (17)

Abbreviations: DM: dry matter, DIM: days in milk, CP: crude protein.

**Table 2:** Influence of days in milk, parity and farm on milk yield and composition and fatty acid concentrations in early lactation Holstein cows.

	DIM (d)			Parity		SEM	*p*-Value		
n	≤50 35	51–99 42	≥100 25	1 39	≥2 57		DIM	Parity	Farm
Milk yield and composition									
Yield (kg/d)	31.33 ^b^	31.5 ^b^	29.00 ^a^	23.62	37.61	0.754	0.01	<0.001	<0.001
Fat (g/kg)	45.00	42.92	44.11	43.92	44.09	0.757	0.19	0.93	0.26
Protein (g/kg)	30.90	31.37	31.77	31.3	31.38	0.317	0.25	0.93	0.03
Fat/protein ratio	1.45 ^b^	1.36 ^a^	1.38 ^a^	1.41	1.38	0.023	0.07	0.54	0.95
Milk FA concentration (% of identified FA)									
C4–C14	19.62 ^b^	21.11 ^a^	21.3 ^a^	19.7	21.64	0.337	0.01	0.03	0.06
16:0	32.81 ^a^	34.02 ^b^	32.55 ^a^	31.78	33.47	0.471	0.02	0.02	0.004
18:0	14.23 ^a^	13.37 ^b^	13.27 ^b^	14.57	12.67	0.229	0.02	0.001	0.02
18:1c9	28.45 ^a^	26.62 ^b^	26.30 ^a,b^	28.12	26.27	0.515	0.01	0.05	<0.001
OBCFA	3.34 ^a^	3.73 ^b^	3.82 ^b^	3.70	3.56	0.054	<0.001	0.13	0.03
PUFA	2.67 ^a^	2.35 ^b^	2.68 ^a^	2.75	2.27	0.054	0.001	0.005	0.007

Data presented as LS means. Abbreviations: N: number of samplings; OBCFA: odd and branched-chain fatty acids; PUFA; polyunsaturated fatty acids. Different superscripts in the same row show statistically different values between DIM groups.

**Table 3:** Influence of days in milk, parity and farm on blood fatty acid concentrations, metabolites and hormones in early lactation Holstein cows.

	DIM (d)			Parity		SEM	*p*-Value		
n	≤50 35	51–99 42	≥100 25	1 39	≥2 57		DIM	Parity	Farm
Body condition score	2.43 ^b^	2.21 ^a^	2.54 ^b^	2.33	2.35	0.036	<0.001	0.62	0.16
Blood metabolites									
Glucose (mg/dL)	60.79 ^a^	60.34 ^a^	50.33 ^b^	57.3	58.34	1.205	0.004	0.66	0.07
Total NEFA (mmol/L)	0.21 ^a^	0.10 ^b^	0.12 ^b^	0.15	0.13	0.018	<0.001	0.43	0.02
BHB (mmol/L)	1.12 ^a^	0.70 ^b^	0.63 ^b^	0.75	0.88	0.059	<0.001	0.11	0.07
Cholesterol (mmol/L)	4.01 ^a^	4.95 ^b^	4.85 ^b^	4.48	4.76	0.147	<0.001	0.36	<0.001
Urea (mmol/L)	3.15 ^a^	3.81 ^b^	4.05 ^b^	3.85	3.62	0.123	<0.001	0.23	<0.001
Triglycerides (mmol/L)	0.11 ^a^	0.13 ^b^	0.15 ^b^	0.13	0.12	0.006	0.004	0.68	0.002
Blood NEFA (% of identified NEFA)									
16:0	24.15	23.67	24.3	23.65	24.80	0.328	0.54	0.02	0.004
18:0	36.94 ^a^	41.68 ^b^	44.48 ^b^	40.58	41.49	0.741	0.004	0.61	0.03
18:1c9	34.98 ^a^	28.38 ^b^	25.4 ^b^	30.61	28.56	0.744	<0.001	0.3	0.005
18:2	3.38 ^a^	6.05 ^b^	5.20 ^b^	4.95	4.39	0.415	<0.001	0.23	0.01
Blood hormones									
Insulin (μUI/mL)	7.44 ^a^	10.27 ^b^	14.29 ^c^	11.27	10.09	0.503	<0.001	0.19	0.02
IGF-I (ng/mL)	30.79 ^a^	58.56 ^b^	88.02 ^c^	63.87	54.38	3.213	<0.001	0.03	0.003

Data presented as LS means. N: number of samplings; NEFA: non esterified fatty acids; BHB: β-hydroxybutyrate; IGF-I: insulin growth factor-I. Different superscripts in the same row show statistically different values between DIM groups.

## Appendix A

**Table A1:** LS means of milk yield and composition and fatty acid concentrations in early lactation Holstein cows in the 5 farms of the study.

Farms						SEM	*p* Value
	1	2	3	4	5		
Milk yield and composition							
Yield (kg/d)	37.83 ^c^	30.8 ^b^	23.78 ^a^	24.88 ^a^	35.81 ^c^	0.754	<0.001
Fat (g/kg)	45.84	40.91	45.49	43.45	44.37	0.757	0.26
Protein (g/kg)	32.53 ^b^	28.92 ^a^	32.58 ^b^	32.18 ^a,b^	30.67 ^a,b^	0.317	0.03
Fat/protein ratio	1.40	1.39	1.43	1.36	1.40	0.023	0.95
Milk FA concentration (% of identified FA)							
C4–C14	21.67 ^b^	20.55 ^a,b^	22.29 ^b^	20.27 ^a,b^	18.60 ^a^	0.03	0.06
16:0	32.28 ^a^	32.96 ^a,b^	37.59 ^b^	31.86 ^a^	30.93 ^a^	0.02	0.004
18:0	12.81 ^a^	13.53 ^a,b^	11.94 ^a^	15.44 ^a,b^	14.40 ^a,b^	0.001	0.02
18:1c9	25.70 ^a^	30.23 ^b^	24.75 ^a^	24.83 ^a^	30.46 ^a,b^	0.05	<0.001
OBCFA	2.71 ^a^	2.51 ^b^	3.82 ^b^	3.70	3.56	0.13	0.03
PUFA	2.71 ^b,c^	2.51 ^b^	2.46 ^b^	2.11 ^a^	3.04 ^c^	0.005	0.007

Data presented as LS means. Abbreviations: N: number of samplings; OBCFA: odd and branched-chain fatty acids; PUFA; polyunsaturated fatty acids. Different superscripts in the same row show statistically different values between DIM groups.

**Table A2:** LS means of blood fatty acid concentrations, metabolites and hormones in early lactation Holstein cows in the 5 farms of the study.

Farms						SEM	*p* Value
	1	2	3	4	5		
Body condition score	2.45	2.25	2.35	2.18	2.38	0.036	0.16
Blood metabolites							
Glucose (mg/dL)	59.36 ^b^	55.04 ^a,b^	58.69 ^b^	66.32 ^b^	49.69 ^a^	1.205	0.07
Total NEFA (mmol/L)	0.24 ^b^	0.13 ^a^	0.11 ^a^	0.11 ^a^	0.13 ^a^	0.018	0.02
BHB (mmol/L)	0.68 ^a^	0.97 ^b^	0.83 ^a,b^	0.68 ^a^	0.92 ^b^	0.059	0.07
Cholesterol (mmol/L)	6.05 ^b^	4.33 ^a^	3.81 ^a^	3.38 ^a^	5.52 ^b^	0.147	<0.001
Urea (mmol/L)	3.78 ^b^	4.61 ^c^	2.19 ^a^	4.31 ^b,c^	3.90 ^b^	0.123	<0.001
Triglycerides (mmol/L)	0.13 ^b^	0.14 ^b^	0.16 ^b^	0.05 ^a^	0.18 ^c^	0.006	0.002
Blood NEFA (% of identified NEFA)							
16:0	23.03 ^a^	25.75 ^b^	24.49 ^a,b^	21.5 ^a^	25.43 ^b^	0.54	0.004
18:0	35.88 ^a^	39.52 ^a,b^	42.77 ^b,c^	48.49 ^c^	38.5 ^a^	0.004	0.03
18:1c9	36.50 ^c^	32.39 ^b,c^	28.36 ^b^	20.31 ^a^	30.38 ^b^	<0.001	0.005
18:2	3.26 ^a^	4.53 ^b^	4.17 ^a,b^	7.00 ^c^	4.39 ^a,b^	<0.001	0.01
Blood hormones							
Insulin (μUI/mL)	9.17 ^a^	8.65 ^a^	11.85^b,c^	13.88 ^c^	9.88 ^a,b^	0.503	0.02
IGF-I (ng/mL)	54.02 ^a^	53.36^a^	55.10^a^	85.99 ^b^	47.16 ^a^	3.213	0.003

Data presented as LS means. N: number of samplings; NEFA: non esterified fatty acids; BHB: β-hydroxybutyrate; IGF-I: insulin growth factor-I. Different superscripts in the same row show statistically different values between DIM groups.
